# Supplementary material for: Mechanical Compression of Human Airway Epithelial Cells Induces Release of Extracellular Vesicles Containing Tenascin C
Source: Cells. 2022 Jan 13;11(2):256. doi: 10.3390/cells11020256 (PMC8774246; doi:10.3390/cells11020256)
Supplement: Supplementary file 1 [file cells-11-00256-s001.zip › cells-1532408-supplementary.pdf]

Table S1. Donor information for primary HBE cells.

Donor	Sex	Age	Ethnicity	BMI	Used in Figure
N1	F	16	Caucasian	19.35	1
N2	F	23	Caucasian	26.18	1
N3	F	59	Hispanic	27.9	1,2,3
N4	F	47	Caucasian	25.1	1,2
N5	M	70	Caucasian	N/A	1, 2,3
N6	M	35	Caucasian	31.42	1,2,3,4
N7	F	39	Hispanic	25.59	2
N8	M	50	Caucasian	27.3	2
A1	F	38	Caucasian	30.9	1
A2	F	28	Caucasian	N/A	1
A3	M	20	Black	N/A	1
A4	F	59	Caucasian	28.5	1

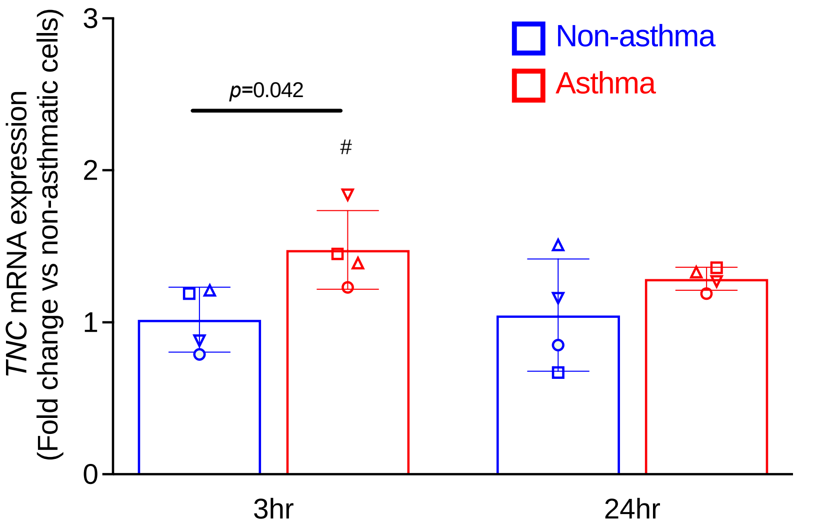

Figure S1. At baseline, *TNC* mRNA expression is marginally higher in asthmatic HBE cells at 3 h. Baseline TNC mRNA expression was measured at 3 h and 24 h in non-asthmatic (mean ± SEM, 4 donors) and asthmatic cells (mean ± SEM, 4 donors). # $p < 0.05$ significantly different between non-asthma and asthma, analyzed by two-way ANOVA with Bonferroni's post-hoc test.

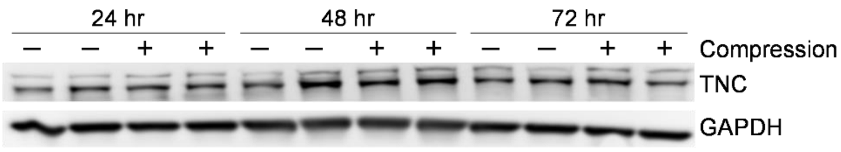

Figure S2. Compression does not increase the cellular expression of TNC. Representative Western blot of three independent experiments showed no change in cellular TNC by mechanical compression. GAPDH was used as a loading control.
